# DNA sequence features underlying large-scale duplications and deletions in human

**DOI:** 10.1007/s13353-022-00704-0

**Published:** 2022-05-20

**Authors:** Mateusz Kołomański, Joanna Szyda, Magdalena Frąszczak, Magda Mielczarek

**Affiliations:** Biostatistics Group, Department of Genetics, Wroclaw University of Environmental and Life Sciences, Wroclaw, Poland

**Keywords:** 1000 Genomes Project, Copy number variants, DNA sequence complexity, GC content

## Abstract

Copy number variants (CNVs) may cover up to 12% of the whole genome and have substantial impact on phenotypes. We used 5867 duplications and 33,181 deletions available from the *1000 Genomes Project* to characterise genomic regions vulnerable to CNV formation and to identify sequence features characteristic for those regions. The GC content for deletions was lower and for duplications was higher than for randomly selected regions. In regions flanking deletions and downstream of duplications, content was higher than in the random sequences, but upstream of duplication content was lower. In duplications and downstream of deletion regions, the percentage of low-complexity sequences was not different from the randomised data. In deletions and upstream of CNVs, it was higher, while for downstream of duplications, it was lower as compared to random sequences. The majority of CNVs intersected with genic regions — mainly with introns. GC content may be associated with CNV formation and CNVs, especially duplications are initiated in low-complexity regions. Moreover, CNVs located or overlapped with introns indicate their role in shaping intron variability. Genic CNV regions were enriched in many essential biological processes such as cell adhesion, synaptic transmission, transport, cytoskeleton organization, immune response and metabolic mechanisms, which indicates that these large-scaled variants play important biological roles.

**Supplementary Information:**

The online version contains supplementary material available at 10.1007/s13353-022-00704-0.

## Introduction

The *1000 Genomes Project*, finished in 2015, resulted in 2504 sequenced genomes of individuals representing 26 populations as well as in the identification of over 88 million of polymorphisms (1000 Genomes Project Consortium et al. 2015). The study found out that an individual human genome differs from the reference genome at 4–5 million sites. The most common type of polymorphisms is single nucleotide polymorphisms (SNPs) — about 84.7 million. Copy number variations (CNVs) defined as deletions and duplications longer than 50 bp are less common that SNPs, but because of their length, they constitute up to 12% of the human genome (Redon et al. 2006). It is known that CNVs are not randomly distributed in eukaryotic genomes, but the biological mechanism of their genomic distribution is not fully understood (Nguyen et al. 2006; Makino et al. 2013). Certainly, there exist a considerable variation in CNV breakpoint location among individuals from the same species, as demonstrated, e.g. by Nicholas et al. (2009) for individuals representing several domestic dog (*Canis familiaris*) breeds. DNA sequence composition is one of the factors triggering the formation of CNV. Repeats of A/T nucleotides and sequences promoting the formation of hairpin structures were observed to mark CNV breakpoints in *Plasmodium falciparum* (Huckaby et al. 2019). Conversely, in mammals (domestic dogs), CNV breakpoints were enriched in G and C nucleotides (Berglund et al. 2012).

The aim of this study was to characterise DNA structure in regions of human genome that are susceptible to structural duplications or deletions. We searched for DNA sequence features promoting the formation of CNVs and the patterns of functional annotations of such deleted and duplicated regions.

## Material and methods

### Dataset

**Fig. 1:**
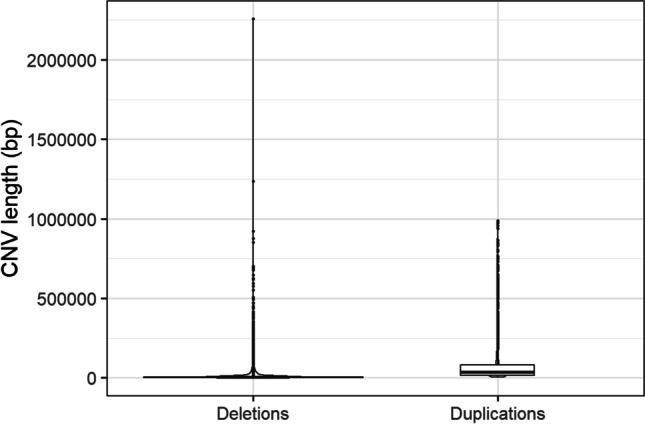
Duplication and deletion length (bp)

The human reference genome GRCh38 was downloaded from the National Center for Biotechnology Information database (NCBI Resource Coordinators 2018). Polymorphisms, including CNVs, were obtained within the frame of the 3rd phase of 1000 Genomes Project and are available from the European Bioinformatics Institute (https://www.ebi.ac.uk) under the ID: *estd214.* Primary data resulted from oligonucleotide genotyping, whole genome and exome sequencing. Nine software packages were used to identify large-scale genomic variants including Breakdancer (Chen et al. 2009), Delly (Rausch et al. 2012), Variation Hunter (Hormozdiari et al. 2010), CNVnator (Abyzov et al. 2011), ReadDepth (Miller et al. 2011), Genome STRIP (Mills et al. 2011), Pindel (Ye et al. 2009), MELT (Gardner et al. 2017) and Dinumt (Dayama et al. 2014) and their call sets were merged. Selected variants were then validated using various methods, including microarrays, PCR-free whole genome sequencing and PacBio sequencing, as well as PCR. The estimated false discovery rate for CNVs was below 5% (1000 Genomes Project Consortium et al. 2015). Since a combination of filtering, calling and validation methods is a recommended approach to obtain reliable large-scale variants (Butty et al. 2020; Gabrielaite et al. 2021), we considered the 1000 Genomes Project Consortium calls as a *high confidence dataset*. In our study, from all available high confidence variants (copy number variants, indels, insertions, inversions and mobile elements), only CNVs defined as duplications or deletions were extracted. Overlapping CNVs were considered independently, resulting in 5867 tandem duplications and 33,181 deletions. Length of duplications ranged between 3006 and 988,090 bp, with median of 37,036 bp and mean of 66,527 ± 91,091 bp. Length of deletions ranged between 204 bp and 2,258,238 bp, with median of 3774 bp and mean of 12,143 ± 34,749 bp (Fig. 1).

### Reference genome sequence features

The Samtools software (Li and Durbin 2009) was used to extract regions covered by CNVs from the GRCh38 reference genome. Moreover, coordinates of reference sequences flanking CNVs (100 nucleotides upstream and downstream of each deletion and duplication) were extracted. These regions were considered in the context of unknown nucleotides (denoted as “N”), Guanine-Cytosine pairs, sequence complexity and functional annotation. In order to compare regions covered by CNVs with random genomic sequences, we selected random region coordinates and extracted the regions from the reference genome, using the Samtools software. The process was repeated to match the actual numbers of analysed CNVs, so that four sets of random sequences were selected: (i) *set 1* contained 5859 sequences of length equal to the median length of duplications (37,036 bp); (ii) *set 2* contained 33,175 sequences of length equal to the median length of deletions (3774 bp); (iii) *set 3* contained 5867 sequences of 100-bp length and was used for comparisons with sequences upstream and downstream of duplications; (iv) *set 4* contained 33,181 sequences of 100-bp length and was used for comparison with sequences upstream and downstream of deletions. All sequences containing unknown nucleotides were excluded. The distributions of GC content were tested for normality using the Kolmogorov test. The H_0_ stating that the distributions of GC content follow the normal distribution with mean and variance given by the considered data sets. The test statistics, which is defined as the supremum of difference between theoretical and empirical distribution, has the same distribution as the classical Kolmogorov statistics. Furthermore, the distributions of GC pair content of high confidence CNV-related sequences were compared with the corresponding randomised sets, i.e. high confidence duplications and set 1, high confidence deletions and set 2, flanking regions of high confidence duplications and set 3, and flanking regions of high confidence deletions and set 4. It was done using the Wilcoxon-Mann–Whitney test, with H_0_ stating that the distributions of GC content are equal. The normalised Wilcoxon-Mann–Whitney test statistic is given by:

$$Z=\frac{V-\frac{n\bullet m}{2}}{\sqrt{\frac{n\bullet m\bullet (n+m+1)}{12}}} \sim N\left(\mathrm{0,1}\right)$$

where $V=\sum_{j=1}^{m}{S}_{j}-\frac{m\bullet (m+1)}{2}$, ${S}_{j}$ denotes ranks corresponding to the GC pair percentage classes in the random sequences, *n* is a count of deletion/duplication/flanking CNV regions and *m* is a count of sets with random sequences. Statistical analysis and figures were done in R package (R Core Team 2013).

### Sequence complexity

Sequence complexity of the entire reference genome was estimated using the sDust software (Morgulis et al. 2006). The overlap between low-complexity regions defined by sDust and CNV-related regions was determined by using the bedtools software (Quinlan and Hall 2010) for high confidence CNVs and flanking regions, as well as for the random sets. The distributions of low-complexity sequence contents in CNV and flanking regions as well as in randomised data were compared the same way as GC pair content by using the Wilcoxon-Mann–Whitney test.

### Functional annotation

The Variant Effect Predictor (VEP) software (McLaren et al. 2016) was used for the functional annotation of CNVs. Gene Ontology enrichment (Mi et al. 2019) was tested using the Fisher’s exact test with the false discovery rate (FDR). Moreover, significantly enriched signalling pathways from the Panther (Mi et al. 2019) database were identified using the KOBAS tool (Xie et al. 2011) applying the Fisher’s exact test with FDR.

## Results

Reference genome sequence features.

### Unknown nucleotide (N) content

Among all of the regions of the human reference genome GRCh38 (Schneider et al. 2017) covered by CNVs, only eight duplications (Supplementary Information S1) and six deletions (Supplementary Information S2) contained unknown nucleotides. Percentage of unknown nucleotides in duplications ranged between 0.002 and 22.06% and in deletions, it varied between 0.0004 and 63.21%. Note that in three deletions, unknown nucleotides constituted over 50% of the whole length. Some regions contained a fixed number of unknown nucleotides (i.e. 100 or 50,000 Ns); what represents the fact that the actual number of unknown nucleotides in the reference cannot be determined. In regions flanking CNVs, only one sequence, located upstream of deletion, contained 17 unknown nucleotides (chromosome: 18, start: 9984, end: 10,083).

### GC content

All sequences containing unknown nucleotides were excluded from the GC content analysis. The average content of GC pairs was very similar in duplications (41.86% ± 5.83) and deletions (41.08% ± 6.15). The lowest content was 29.08% in duplications and 21.27% in deletions, while the maximum contents were respectively 68.90% and 73.46% (Table 1). The visual examination of GC pair content distributions in CNVs, presented on Figs. S3a and S4a in the supplementary material, demonstrated that both are skewed indicating an excess of low GC contents, while the regions flanking CNVs exhibit a more symmetric distribution (Figs. S5a, S5b, S6a and S6b), indicating no link between CNV breakpoint formation and the GC content. The distributions of GC pair contents of duplications (*P* = 0.004) and deletions (*P* = 7.955·10^−12^) significantly differed from the contents of corresponding randomised sequences. In particular, high confidence deletions contained less GC pairs than random regions (*P* = 3.977·10^−12^), while duplications were enriched in GC pairs as compared to a randomised set of sequences (*P* = 0.0024). High confidence deletion flanking regions contained more GC pairs than the corresponding randomised sequences, i.e. *P* = 1.5·10^−10^ for upstream and *P* = 1.259·10^−21^ for downstream regions. The same situation was observed for downstream region of duplications (*P* = 1.74·10^−9^), but for upstream, it was lower than in random case (*P* = 0.014). graphical representation of Randomised duplications (Fig. S3b), deletions (Fig. S4b) and their flanking regions (Figs. S5c and S6c) GC content distributions are provided in the supplementary material.

**Table 1:** Guanine-cytosine pair content (%) in the investigated regions

Region	Min	Mean	Max	Sd
**Duplications**	**29.08**	**41.86**	**68.90**	**5.83**
Set 1 (randomised duplications)	31.74	41.59	65.74	5.63
Upstream duplications	7.00	41.24	83.00	11.59
Downstream duplications	6.00	42.60	86.00	10.73
Set 3 (randomised upstream and downstream duplications)	1.00	41.42	84.00	10.54
**Deletions**	**21.27**	**41.08**	**73.46**	**6.15**
Set 2 (randomised deletions)	20.56	41.54	77.19	6.53
Upstream deletions	0.00	41.82	84.00	10.53
Downstream deletions	0.00	42.05	89.00	10.47
Set 4 (randomised upstream and downstream deletions)	0.00	41.41	89.00	10.66

### Sequence complexity

**Fig. 2:**
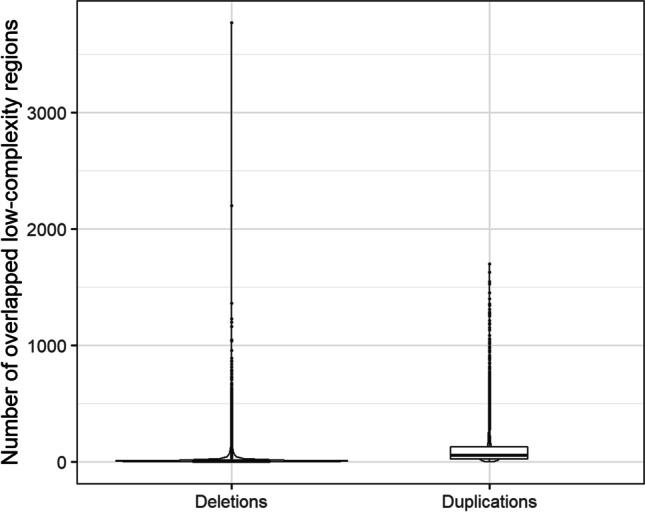
The number of LCRs overlapped duplications and deletions

**Fig. 3:**
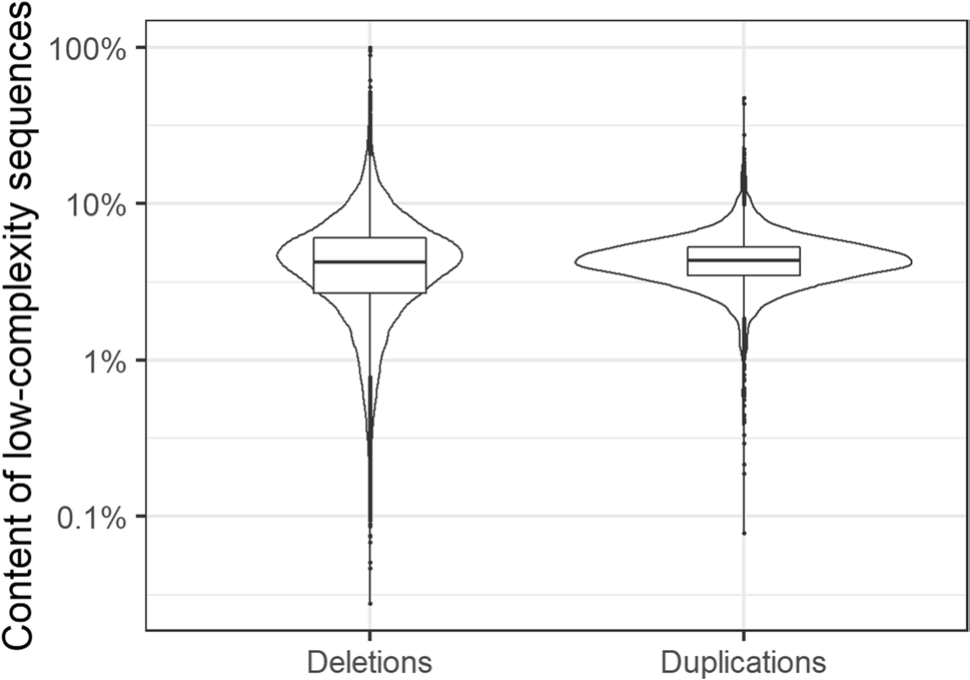
Low-complexity sequence content in duplications and deletions (CNVs not overlapping any region are not presented)

A total of 4,798,406 low-complexity regions (LCRs) were identified within the whole GRCh38 reference genome. Lengths of those regions varied between 7 and 25,072 bp with mean of 29 bp (± 56). All duplications and 93.93% of deletions contained within LCRs. Median number of LCRs overlapped with a single duplication was 57 and with a single deletion was six (Fig. 2, Table 2). On average, LCRs made up 4.59% of a duplication length and 4.66% of a deletion length (Fig. 3, Table 2). On the other hand, CNV breakpoint regions contained much less LCRs. Only, 20.83% of sequences upstream of duplications and 16.52% of sequences downstream of duplications contained a low-complexity region(s). Similarly among deletion breakpoints, we identified 20.37% of upstream sequences and 19.25% of downstream sequences with LCR. Among them, on average, 4.73% of the length of regions upstream of duplications, 3.47% of the length of regions downstream of duplications, 4.44% of the length of regions located upstream of deletions and 4.16% of the length of regions downstream of deletions. In the random sequence set 1, 99.21% of sequences contained low-complexity regions, in set 2 — 97.62%, in set 3 — 18.12% and in set 4 — 18.61%. None of the empirically constructed frequency distributions in the considered regions deviated from the normal distribution. The distributions of low-complexity sequence content in randomised duplications and in high confidence duplications (*P* = 0.106) as well as between randomised downstream deletions and regions downstream of deletions (*P* = 0.078) did not differ. The percentage of low-complexity sequences was significantly higher upstream of deletions (*P* = 1.907·10^−8^), upstream of duplications (*P* = 8.982·10^−5^) and within deletions (*P* = 2.963·10^−19^) than in corresponding randomised upstream and downstream regions. Conversely, the distribution of low-complexity sequence content downstream of duplications was significantly lower than in set 3 (*P* = 0.007).

**Table 2:** Content of low-complexity regions (LCR) within CNV-related regions

Regions	Number of overlapped LCRs			Content of LCRs (%)		
	Min	Mean	Max	Min	Mean	Max
**Duplications**	**1**	**104**	**1 698**	**0.07**	**4.59**	**47.52**
Set 1 (randomised duplications)	0	58	114	0.00	4.49	59.44
Upstream duplications	0	0	3	0.00	4.73	100.00
Downstream duplications	0	0	2	0.00	3.47	100.00
Set 3 (randomised upstream and downstream duplications)	0	0	3	0.00	4.19	100.00
**Deletions**	**0**	**6**	**3 769**	**0.00**	**4.66**	**100.00**
Set 2 (randomised deletions)	0	6	25	0.00	4.55	98.07
Upstream deletions	0	0	3	0.00	4.44	100.00
Downstream deletions	0	0	3	0.00	4.16	100.00
Set 4 (randomised upstream and downstream deletions)	0	0	4	0.00	4.36	100.00

### Functional annotation of CNVs

The 5867 duplications overlapped with 9111 genes corresponding to 35,317 transcripts. The 33,181 deletions overlapped with 19,022 genes corresponding to 71,542 transcripts. The most common Sequence Ontology (SO) terms determined for **duplications** comprised intron variants (SO:0,001,627) and transcript amplifications (SO:0,001,889) (Table 3). For **deletions**, the most common SO terms were as follows: feature truncations (SO:0,001,906) and intron variants (SO:0,001,627) (Table 3). In the context of biological processes, genes containing duplications were significantly overrepresented in ontologies related to homophilic cell adhesion via plasma membrane adhesion molecules (GO:0,007,156), modulation of chemical synaptic transmission (GO:0,050,804), cytoskeleton organization (GO:0,007,010) and in the Cadherin signalling pathway (P00012) as well as underrepresented for complement activation, classical pathway (GO:0,006,958), including immune response (GO:0,006,955). Genes containing deletions were significantly overrepresented in GO terms related to transport (GO:0,006,810), cellular component organization (GO:0,016,043) and regulation of cellular processes (GO:0,050,794).

**Table 3:** Functional annotation of duplications and deletions

Consequences for duplications	SO accession	Percent of variants
Intron variant	SO:0,001,627	25
Transcript amplification	SO:0,001,889	24
Coding sequence variant	SO:0,001,580	12
Feature elongation	SO:0,001,907	8
Non-coding transcript exon variant	SO:0,001,792	8
5-prime UTR variant	SO:0,001,623	7
3-prime UTR variant	SO:0,001,624	6
Upstream gene variant	SO:0,001,631	3
Downstream gene variant	SO:0,001,632	3
NMD transcript variant	SO:0,001,621	2
Non-coding transcript variant	SO:0,001,619	1
Other	-	1
**Consequences for deletions**	**SO accession**	**Percent of variants**
Feature truncation	SO:0,001,906	31
Intron variant	SO:0,001,627	31
Non-coding transcript variant	SO:0,001,619	10
Upstream gene variant	SO:0,001,631	4
Non-coding transcript exon variant	SO:0,001,792	4
Downstream gene variant	SO:0,001,632	4
Transcript ablation	SO:0,001,893	4
Intergenic variant	SO:0,001,628	3
Coding sequence variant	SO:0,001,580	3
NMD transcript variant	SO:0,001,621	3
5-prime UTR variant	SO:0,001,623	1
3-prime UTR variant	SO:0,001,624	1
Stop lost	SO:0,001,578	1

## Discussion

Our study revealed a non-random distribution of GC pairs within CNVs and in CNV flanking regions. This could have been expected, following the hypothesis that the GC content serves as a tool for differentiation between intron (lower GC content) and exons (higher GC content) during splicing (Amit et al. 2012). In our study, we observed that the GC content of deletions was lower and of duplications — higher than in random genomic regions, what indicates that intronic regions are more prone to deletions, whereas exonic regions are more prone to duplications. However, a contradictory result was obtained for humans by Rigau et al. (2019) who observed that deleted regions had significantly higher GC content. In our study, the majority of deletions was annotated to introns what further supports the GC content imbalance (Aïssani and Bernardi 1991). Deletions in genic regions, containing more GC, are functionally more severe than duplications. Moreover, duplications which associate with GC-rich regions (i.e. exons) have some evolutionary advantage (Levasseur and Pontarotti 2011). It is also worth to notice that according to Dittwald et al. (2013), GC content is positively correlated with the frequency of nonallelic homologous recombination (NAHR) which is a common cause of CNV formation. According to Romiguier et al. (2010), GC-rich sequences are prone to deletions because base composition imbalance triggers replication slippage. On the other hand, Chen et al. (2011) did not report a difference in GC content between CNV regions and autosomal average. Our study also demonstrated a non-random GC content in CNV flanking regions, albeit without a consistent trend, i.e. enriched GC content in deletion breakpoints, but only downstream of duplications. Similarly, Bose et al. (2014) investigated SNV breakpoints and concluded that all SV types had a higher GC percentage than the genome average.

Also in terms of sequence complexity, a non-random pattern was revealed, with deletions being enriched with LCR, but without a consistent pattern in breakpoint regions. Barski et al. (2019) investigated sequence complexity in regions flanking CNV in *Bos taurus*. The study concluded that duplications and deletions preferentially form in regions of low complexity. CNVs also appear to be enriched in regions of low mappability, as well as within satellites and Short Tandem Repeats (Nguyen et al. 2006; Monlong et al. 2018), all of those characterised by low complexity. Chen et al. (2014) postulated that low-copy and high-copy repeats can induce DNA instability, resulting in errors in replication and repair mechanisms and consequently leading to the formation of CNVs.

Functional annotation revealed that majority of CNVs were located in introns. Similar observation was made by Chen et al. (2011) for population-specific CNVs. Higher gene density in regions covered by CNVs than in random genome regions was also highlighted by Johansson and Feuk (2011). Moreover, Nguyen et al. (2006) reasoned that large-scale DNA changes, if beneficial, they should be enriched in genes, especially those involved in fighting infection and sensing our environment. According to Rigau et al. (2019), intronic deletions are the most frequent CNVs in protein-coding genes in humans, while deletions overlapping exons are less frequent than expected by chance. Therefore, it was also suggested that intronic CNVs contribute to the variability of gene expression and splicing in human populations. The homophilic cell adhesion identified as an ontology over-represented in deletions in our study was also reported for genes with somatic duplications in placenta by Kasak et al. (2015). Moreover, Morello et al. (2019) observed that synaptic transmission, an ontology over-represented in deletions in our study, was the most highly enriched term in CNV-driven differentially expressed genes in a sporadic form of amyotrophic lateral sclerosis. Involvement of CNVs in immune response mechanisms has already been reported by Perry et al. (2008) and, the same as in our study, genes with immune response functions were overrepresented in human CNV regions (Redon et al. 2006). Deleted genes were significantly overrepresented in GO terms related to transport, cellular component organization and regulation of cellular process, which indicates that deletions significantly affect essential cellular mechanisms (Alloza et al. 2011). Duplicated genes were enriched in the Cadherin signalling pathway, which is involved in multiple biological processes, such as development, neurogenesis, cell adhesion and inflammation. Its enrichment has been reported in the context of many diseases including cancer (Mi et al. 2019).

In conclusions, genomic regions containing large-scale duplications and deletions, called copy number variants (CNVs), constitute a common source of genetic variation. In this study, we analysed duplications and deletions identified within the frame of the 1000 Genomes Project, in the context of identification of the unique DNA sequence features in CNV regions and of annotation of CNVs to functional segments of the human genome. We discovered that (i) guanine-cytosine content is associated with the formation of CNVs; (ii) duplications are initiated in low-complexity regions and (iii) CNVs are preferentially located within introns. Our findings provide a step towards more complete understanding of the human genomic landscape in the context of copy number variants.

## Supplementary Information

Below is the link to the electronic supplementary material.

Supplementary file1 (DOCX 18 KB)

Supplementary file2 (DOCX 215 KB)

## Data Availability

All data were downloaded from the publicly available databases.
