# Supplementary material for: DNA sequence features underlying large-scale duplications and deletions in human
Source: J Appl Genet. 2022 May 20;63(3):527–33. doi: 10.1007/s13353-022-00704-0 (PMC9365719; doi:10.1007/s13353-022-00704-0)
Supplement: Supplementary file 1 — Supplementary file1 (DOCX 18 KB) [file 13353_2022_704_MOESM1_ESM.docx]

Table S1. Unknown nucleotide (N) content in duplicated regions

| **Chromosome** | **Start** | **End** | **Length** | **N-content** |
| --- | --- | --- | --- | --- |
| 2 | 32 403 872 | 33 107 363 | 703 491 | 2 000 (0.28%) |
| 6 | 167 529 394 | 167 756 083 | 226 689 | 50 000 (22.06%) |
| 7 | 136 433 | 405 502 | 269 069 | 2 396 (0.89%) |
| 10 | 53 027 785 | 53 147 359 | 119 574 | 2 (0.002%) |
| 10 | 131 249 138 | 132 223 150 | 974 012 | 100 (0.01%) |
| 10 | 131 446 890 | 131 640 975 | 194 085 | 100 (0.05%) |
| 17 | 425 092 | 895 536 | 470 444 | 41 515 (8.82%) |
| 22 | 49 608 227 | 49 641 383 | 33 156 | 1 (0.003%) |

Table S2. Unknown nucleotide (N) content in deleted regions

| **Chromosome** | **Start** | **End** | **Length** | **N-content** |
| --- | --- | --- | --- | --- |
| 3 | 59 888 393 | 60 159 064 | 270 671 | 1 (0.0004%) |
| 5 | 17 508 562 | 17 596 059 | 87 497 | 50 000 (57.14%) |
| 5 | 17 526 780 | 17 605 880 | 79 100 | 50 000 (63.21%) |
| 8 | 7 227 940 | 7 680 412 | 452 472 | 50 000 (11.05%) |
| 10 | 38 529 525 | 38 614 773 | 85 248 | 43 431 (50.95%) |
| X | 155 916 731 | 155 919 319 | 2 588 | 1 (0.04%) |
