# Supplementary material for: DNA sequence features underlying large-scale duplications and deletions in human
Source: J Appl Genet. 2022 May 20;63(3):527–33. doi: 10.1007/s13353-022-00704-0 (PMC9365719; doi:10.1007/s13353-022-00704-0)
Supplement: Supplementary file 2 — Supplementary file2 (DOCX 215 KB) [file 13353_2022_704_MOESM2_ESM.docx]

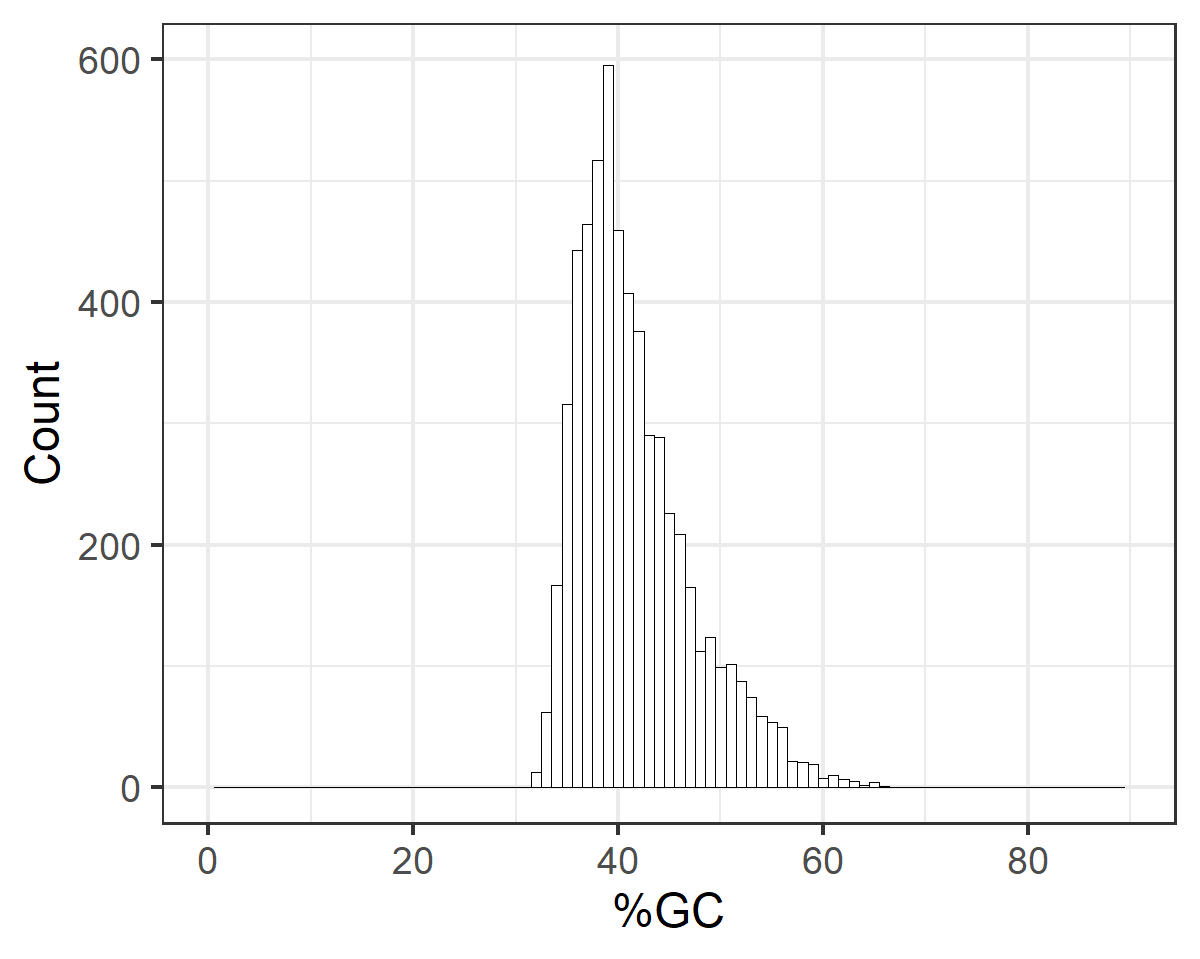

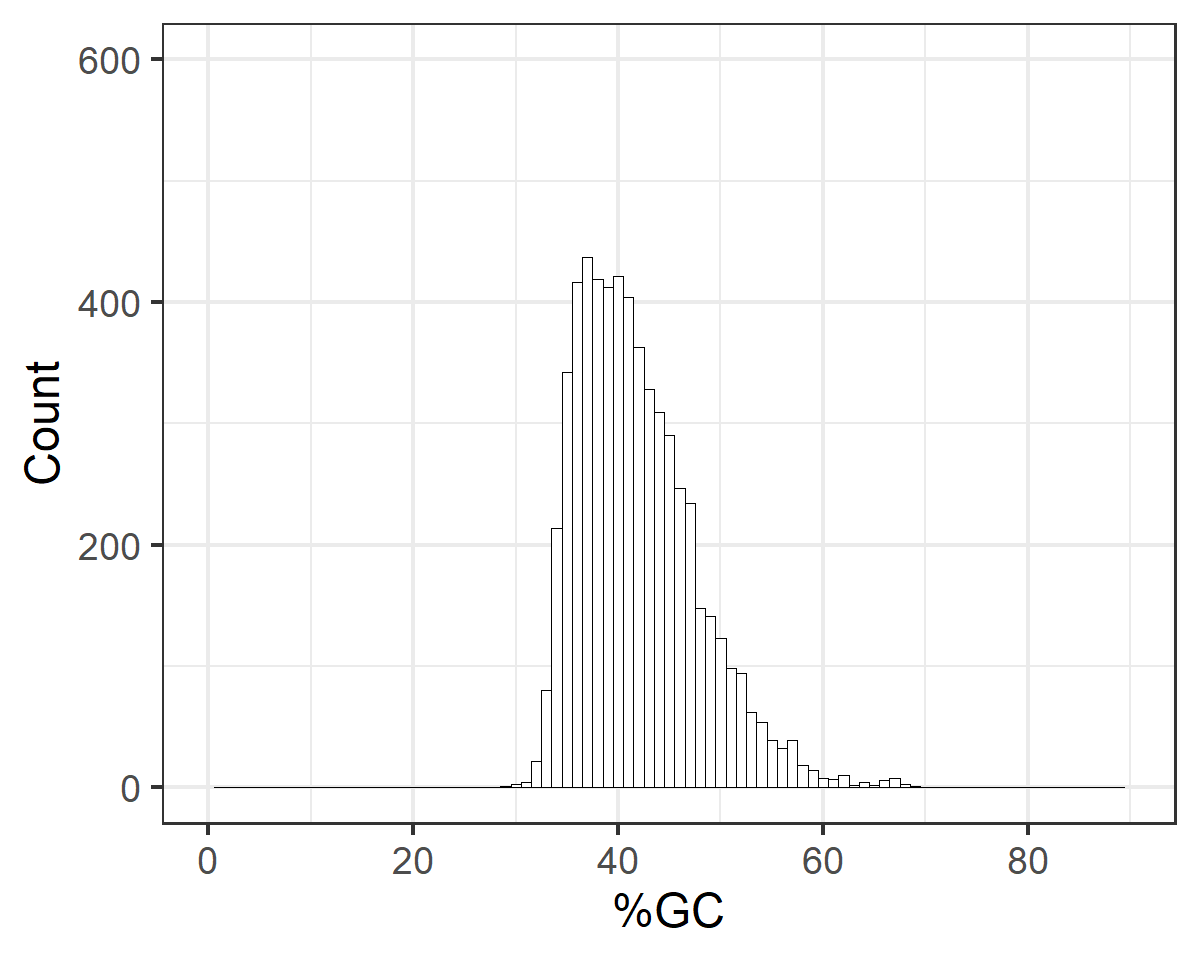


**a**

**b**

Figure S3. Percent of GC pairs for all duplications (a) and percent of GC pairs in Set 1 representing randomised duplications (b).


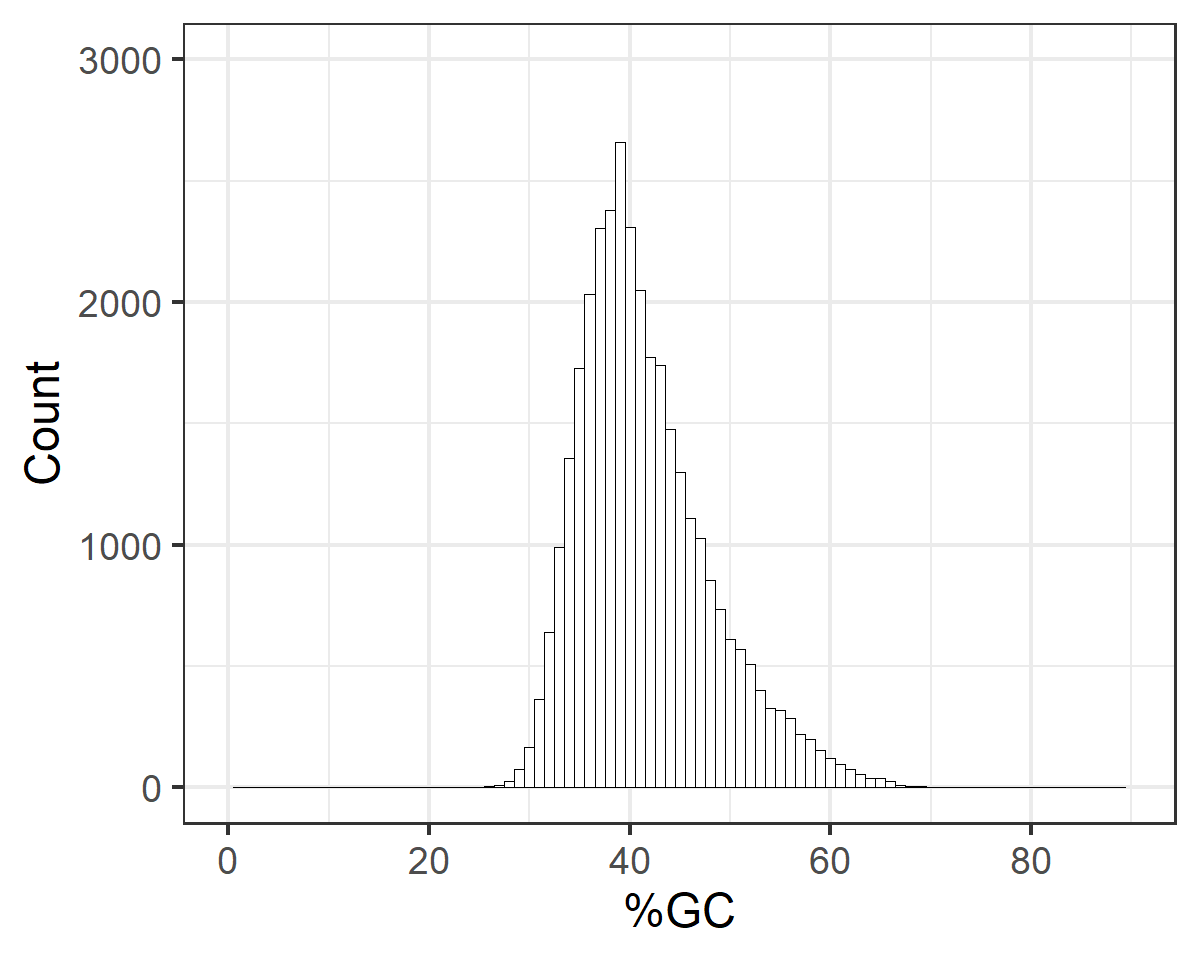

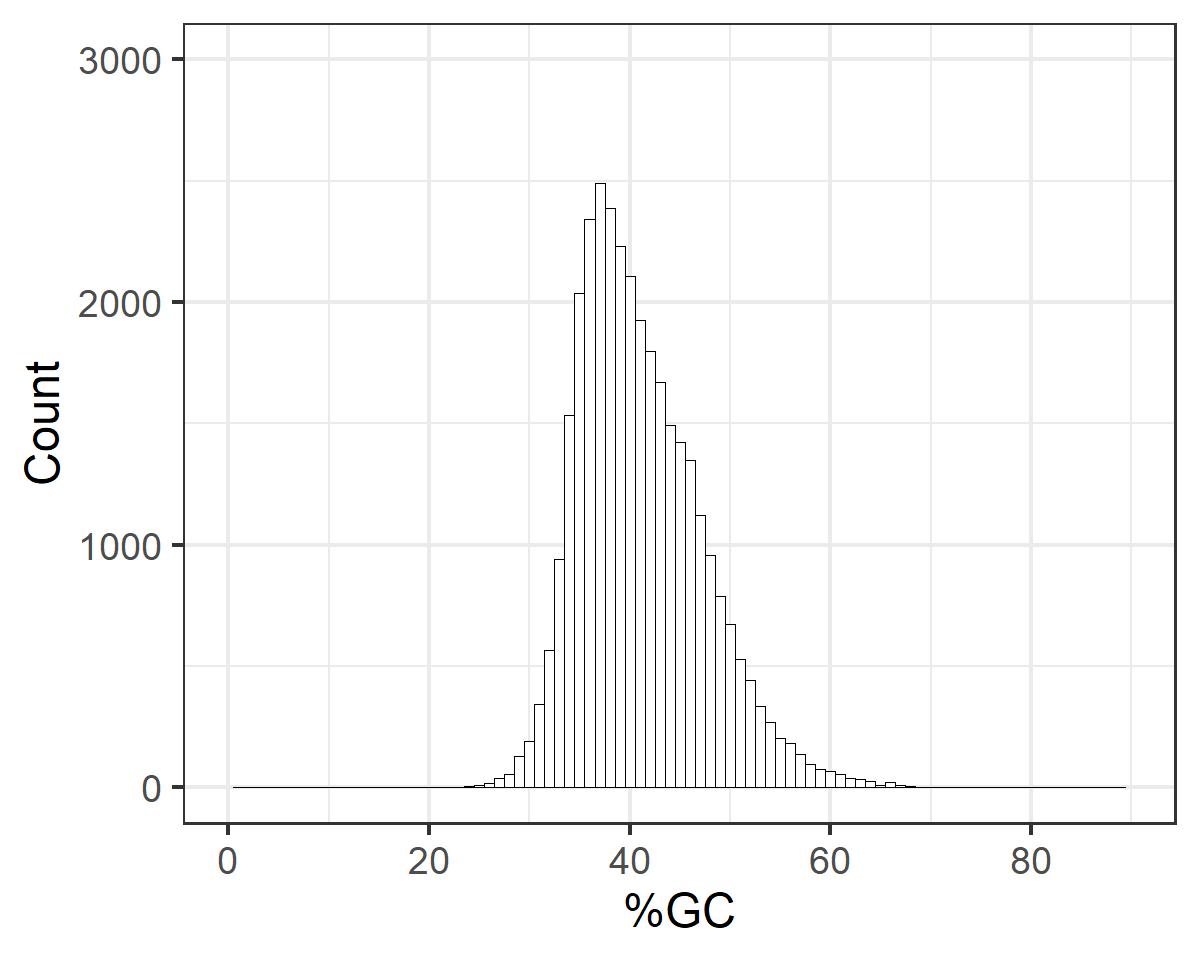


**a**

**b**

Figure S4. Percent of GC pairs for all deletions (a) and percent of GC pairs in Set 2 representing randomised deletions (b).


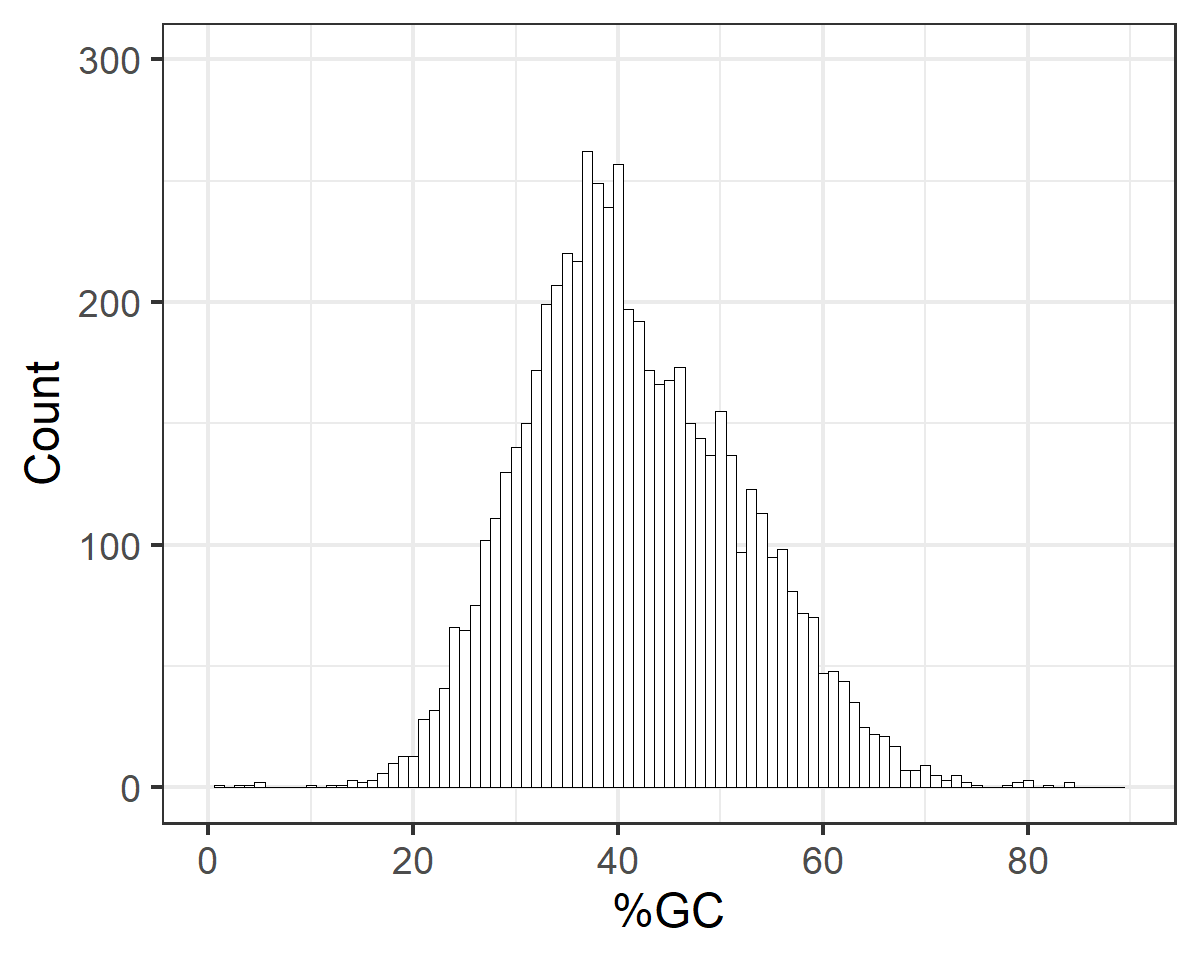

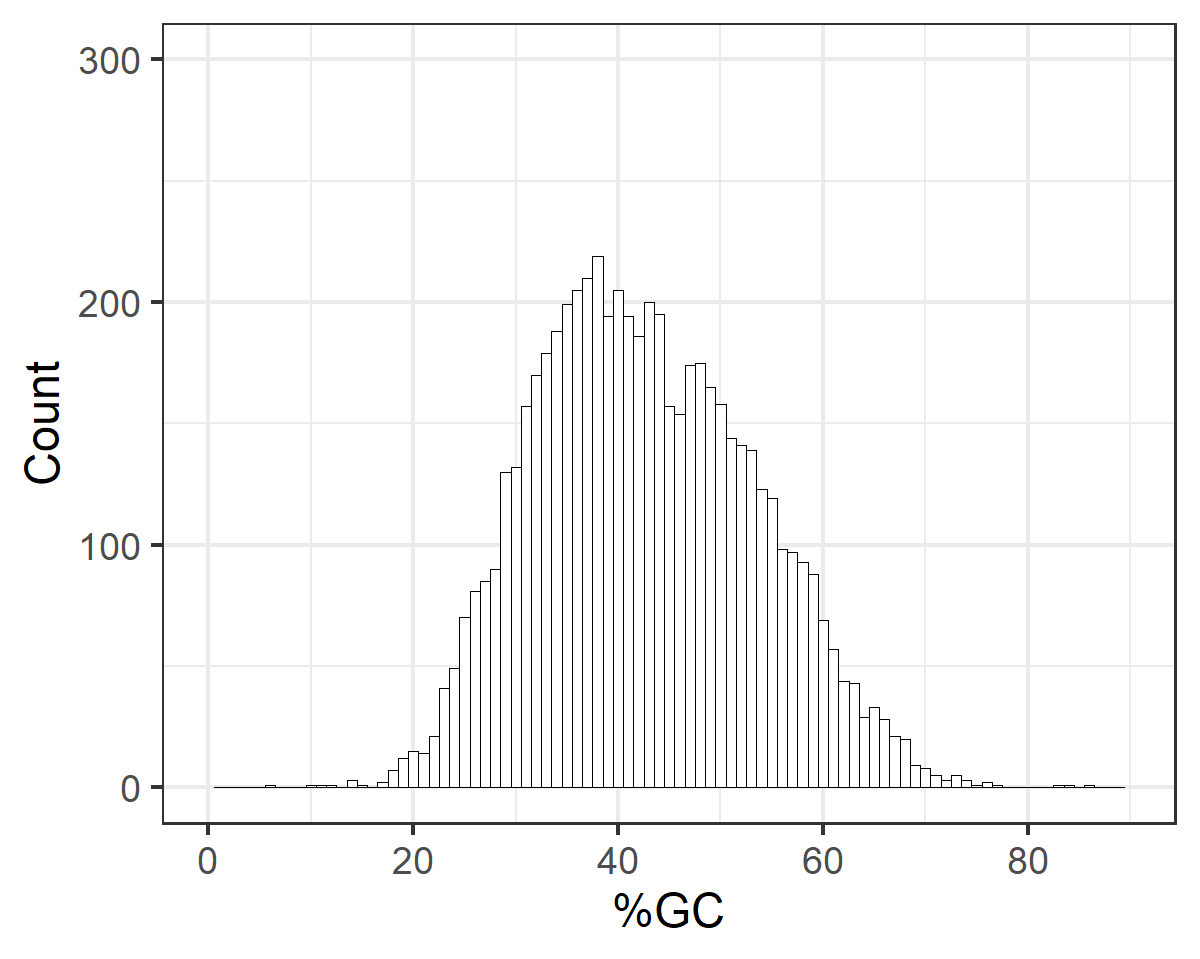

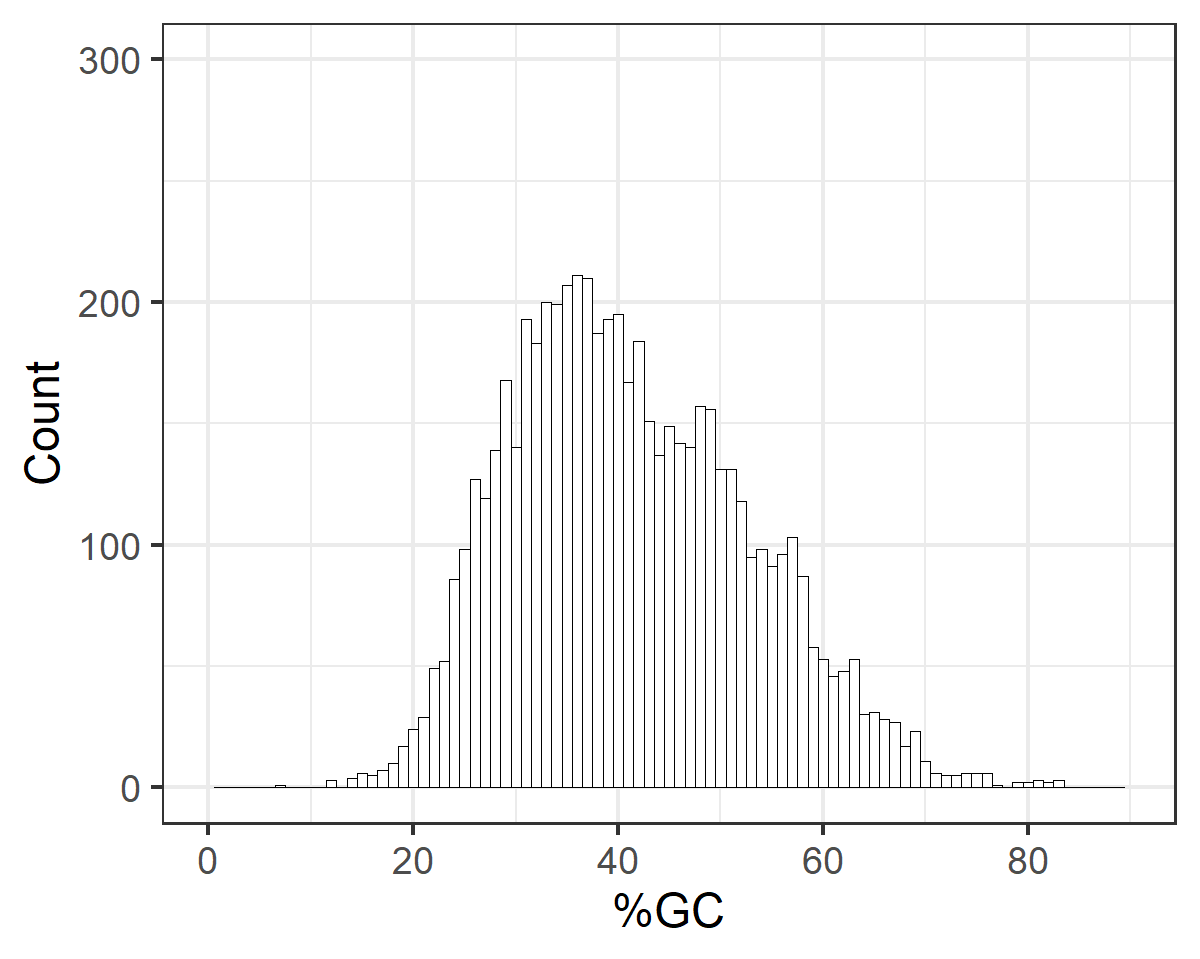


Figure S5. Percent of GC pairs in 100 nucleotides long sequences located (a) up-, (b) downstream duplications and (c) randomised set of 100 nucleotides long sequences representing Set 3.

**a**

**c**

**b**


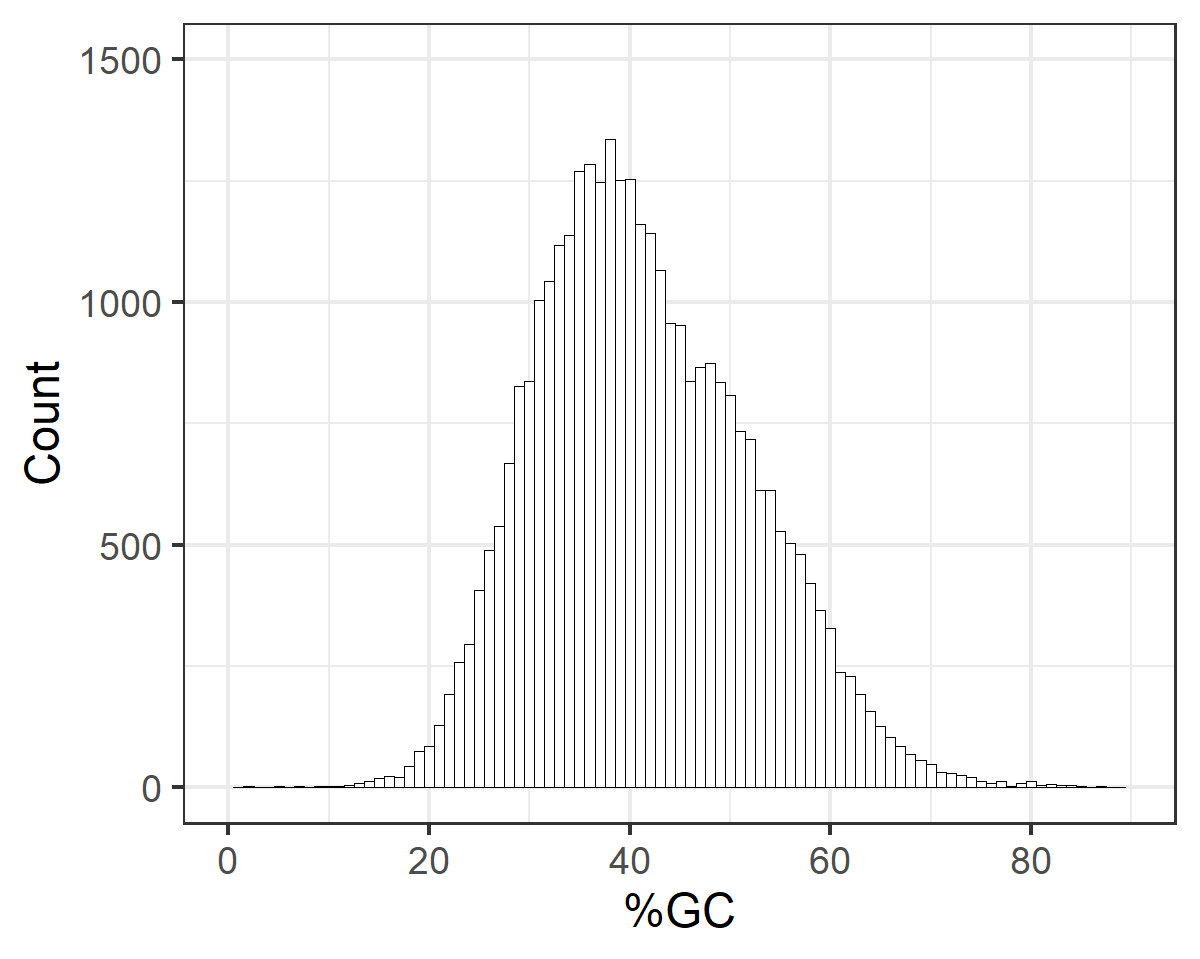

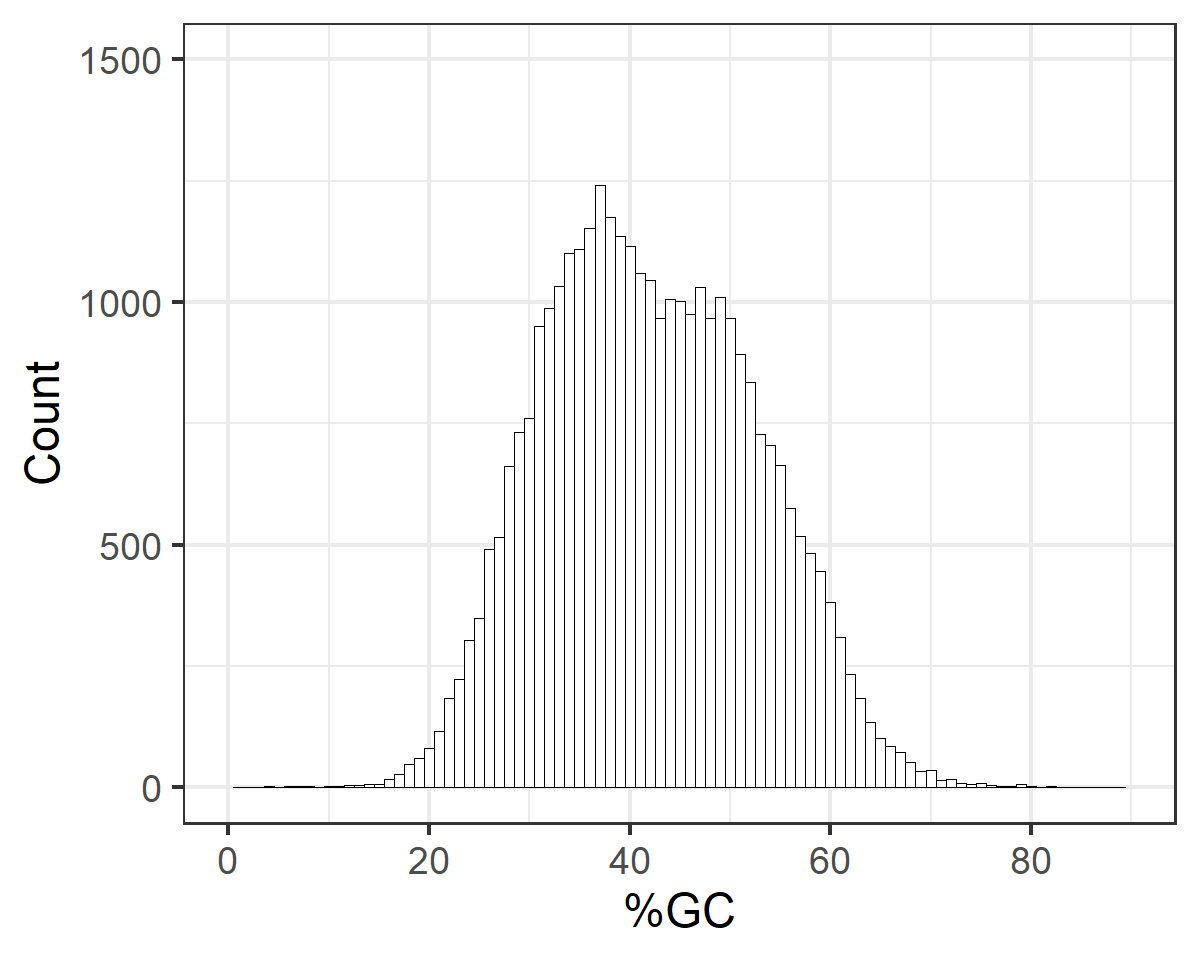

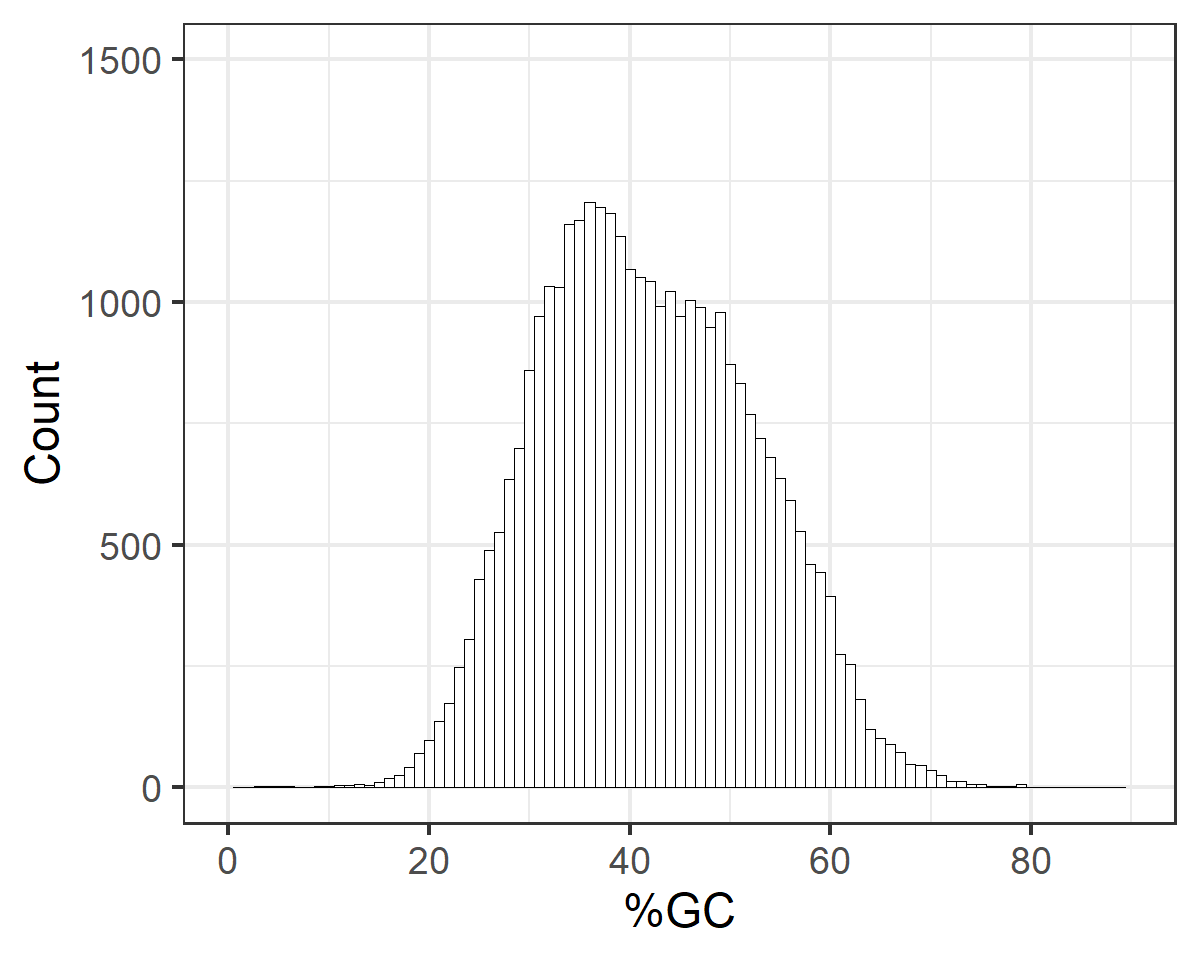


**c**

**b**

**a**

Figure S6. Percent of GC pairs in 100 nucleotides long sequences located (a) up-, (b) downstream deletions and (c) randomised set of 100 nucleotides long sequences representing Set 4.
